# Longitudinal phenotype development in a minipig model of neurofibromatosis type 1

**DOI:** 10.1038/s41598-020-61251-4

**Published:** 2020-03-19

**Authors:** Johanna Uthoff, Jared Larson, Takashi S. Sato, Emily Hammond, Kimberly E. Schroeder, Frank Rohret, Christopher S. Rogers, Dawn E. Quelle, Benjamin W. Darbro, Rajesh Khanna, Jill M. Weimer, David K. Meyerholz, Jessica C. Sieren

**Affiliations:** 1 Department of Radiology, University of Iowa, Iowa City, IA USA; 2 Department of Biomedical Engineering, University of Iowa, Iowa City, IA USA; 3 Holden Comprehensive Cancer Center, University of Iowa, Iowa City, IA USA; 4 Exemplar Genetics, Coralville, IA USA; 5 Department of Pharmacology, University of Iowa, Iowa City, IA USA; 6 Department of Pediatrics, University of Iowa, Iowa City, IA USA; 7 Department of Pharmacology, University of Arizona, Tucson, AZ USA; 8 Department of Pathology, University of Iowa, Iowa City, IA USA; 9 Pediatrics and Rare Diseases Group, Sanford Research, Sioux Falls, SD USA

**Keywords:** Neurodegenerative diseases, Experimental models of disease, Translational research

## Abstract

Neurofibromatosis type 1 (NF1) is a rare, autosomal dominant disease with variable clinical presentations. Large animal models are useful to help dissect molecular mechanisms, determine relevant biomarkers, and develop effective therapeutics. Here, we studied a NF1 minipig model (*NF1*^*+/ex42del*^) for the first 12 months of life to evaluate phenotype development, track disease progression, and provide a comparison to human subjects. Through systematic evaluation, we have shown that compared to littermate controls, the NF1 model develops phenotypic characteristics of human NF1: [1] café-au-lait macules, [2] axillary/inguinal freckling, [3] shortened stature, [4] tibial bone curvature, and [5] neurofibroma. At 4 months, full body computed tomography imaging detected significantly smaller long bones in *NF1*^*+/ex42del*^ minipigs compared to controls, indicative of shorter stature. We found quantitative evidence of tibial bowing in a subpopulation of NF1 minipigs. By 8 months, an *NF1*^*+/ex42del*^ boar developed a large diffuse shoulder neurofibroma, visualized on magnetic resonance imaging, which subsequently grew in size and depth as the animal aged up to 20 months. The *NF1*^*+/ex42del*^ minipig model progressively demonstrates signature attributes that parallel clinical manifestations seen in humans and provides a viable tool for future translational NF1 research.

## Introduction

Neurofibromatosis type 1 (NF1) is an autosomal dominant disorder with nearly 100% penetrance and an incidence of approximately 1 in every 3,000 births worldwide^1^. The clinical manifestations of the disorder are highly variable, even among individuals with the same mutation in NF1^1,2^. The most common phenotypic presentations of NF1 include café-au-lait macules (CALMs), axillary and inguinal freckling, optic pathway gliomas, pilocytic astrocytoma, and the presence of neurofibromas and plexiform neurofibromas (PNs) that have the potential to develop into malignant peripheral nerve sheath tumors (MPNSTs)^1–3^. Some individuals with NF1 may exhibit learning disabilities, increased pain, short stature, or macrocephaly at birth^3,4^. Although uncommon, they may also be born with or develop tibial bowing and sphenoid wing dysplasia^4^. Those affected by NF1 are also at increased risk of developing a number of different cancers including glioblastoma, breast cancer, and leukemia^1^. Because of the heterogeneity, impact on quality of life, and significant morbidity associated with NF1, there is a considerable need for research into its underlying pathology and noninvasive monitoring of its progression.

Systematic study of NF1 in human subjects is a major challenge as the variability in disease presentation between individuals makes it difficult to create an effective longitudinal cohort. Medical imaging is used in the NF1 population to detect and monitor disease phenotypes including optic-pathway glioma^5–7^, plexiform neurofibroma and MPNST, spinal neurofibroma, tibial bowing and scoliosis. Retrospectively collected imaging studies have assessed prevalence and NF1 findings in radiograph^8^, computed tomography (CT)^9^, magnetic resonance (MR) imaging^7,10,11^, and positron emission tomography (PET)^12^. As the NF1 population is more susceptible to the effects of radiation^13^, prospective studies are often limited to non-ionization radiation modalities such as MR^5,6,14,15^ and ultrasound^16,17^.

Efforts to create animal models of NF1 seek to provide the opportunity to explore treatment response and natural course^18–24^. Several murine models of NF1 have already been developed; however, mice are too small for the relevant medical imaging systems to be used in detection and monitoring the progression of NF1’s phenotypes^19–21,24^. Minipigs have proven to be successful models in a range of human diseases including Huntington’s disease, ataxia-telangiectasia, cystic fibrosis, and cancer^25–33^. The similarities between the minipig and humans with regards to anatomy, physiology, and size allows them to be studied using the same imaging systems that are used clinically for human patients. Our previous work with minipigs has shown that a deletion of exon 42 (*NF1*^*+/ex42del*^), a mutation observed in NF1 patients, mimics early in life, a wide range of phenotypes exhibited by the human population including CALMs, cutaneous and/or plexiform neurofibromas, axillary and inguinal freckling, unidentified bright objects, decreased cognitive abilities^22^, and increased pain and sleep disturbances^34^. In this study, we examine and monitor the development of NF1 characteristics in a second generation of Yucatan minipig using CT and MR over the first 12-months of life. Image time points at 4, 8, and 12 months target key developmental stages corresponding to pre-puberty, adolescence and young adulthood in the pig^35,36^. This study provided a unique opportunity for systematic evaluation to capture the natural penetrance of phenotypical presentation and track progression of disease manifestation of *NF1*^*+/ex42del*^ minipigs compared to sibling wildtype controls.

## Results

A flowchart of the study cohort across the repeated imaging timepoints (TPs) is included as Fig. S1 with subject-level indication of phenotype development.

### NF1 minipigs exhibit stable café-au-lait macules and axillary/inguinal freckling

**Figure 1:**
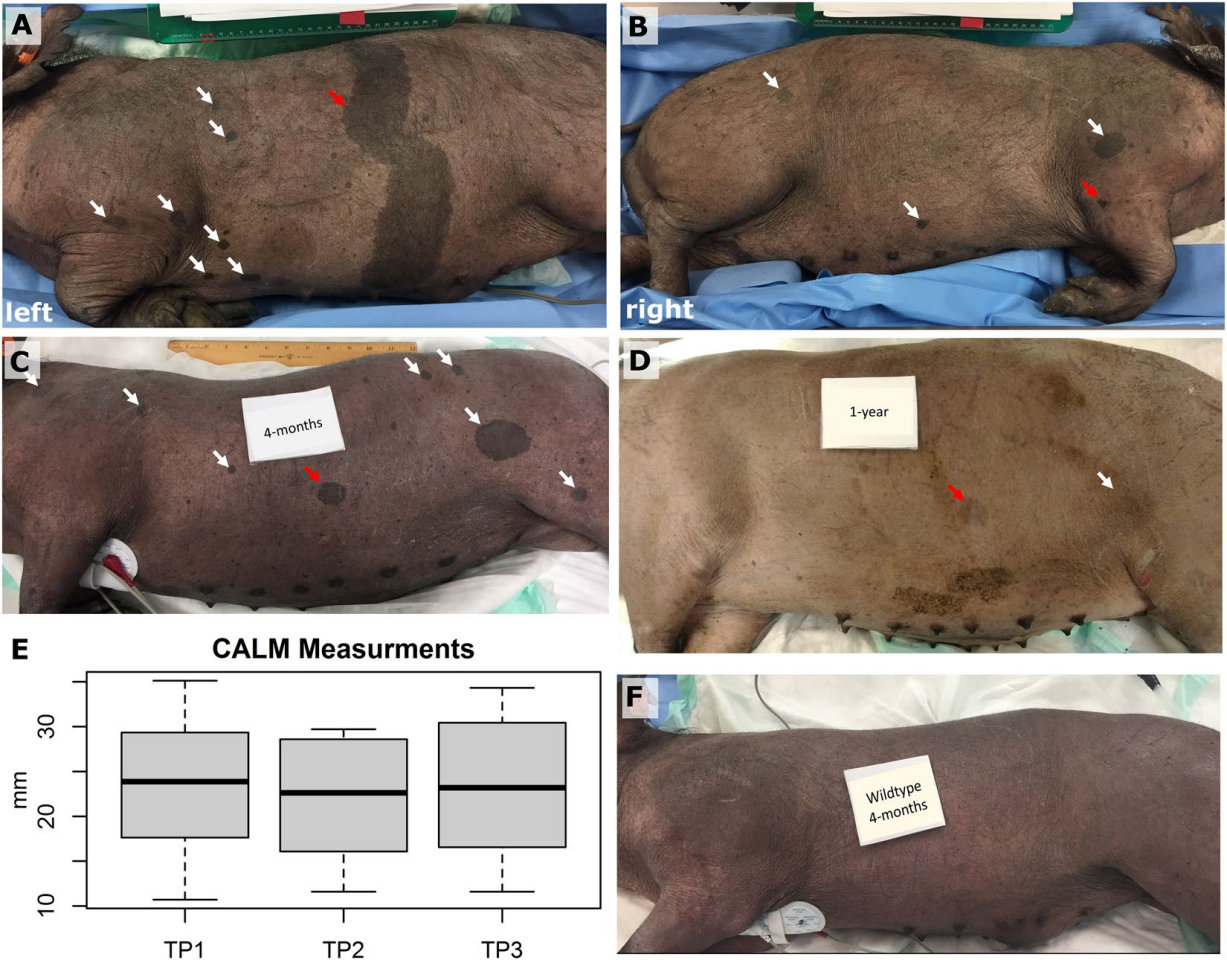
Café au lait macule (CALM) findings. All *NF1*^*+/ex42del*^ minipigs presented with mutilple CALMs (in all pannels arrows: white –visually tracked, red – longtiudinal measurment). (**A**) Left side of *NF1*^*+/ex42del*^ subject at TP1 (aged 4-months) presenting with mutiple CALMs (white arrow) and one large marking (red arrow) spanning the dorsal-ventral side. (**B**) Right side of same subject shows more CALMs. (**C**) Left side of *NF1*^*+/ex42del*^ subject at TP1 (aged 4-months) presenting with mutiple CALMs. (**D**) Left side of same animal at TP3 demonstrates notable skin and CALM lightening with certain markings (white) being undetectable on photograph. (**E**) Boxplot of longtiudinal measurment of CALM markings shows relative stability in marking size (N = 8). (**F**) Left side of wildtype littermate control at TP1 (4-months) presenting without evidence of CALM.

All eight *NF1*^*+/ex42del*^ subjects presented with multiple CALMs at the first imaging timepoint (TP1, 4 months of age), while no CALMs were present on the four wildtype subjects (Fig. 1, Fig. S2**)**. One large marking (Fig. 1A) was encountered which spanned the dorsal-ventral length of the left side in the subject who later developed a left-sided neurofibroma. The selected lateral CALMs ranged from 10.7 mm to 47.8 mm in maximum diameter (Fig. 1A–D**)**. No quantitative evidence of CALM growth over time was found in subjects over the three timepoints (Fig. 1E) or in the wildtype subjects (Fig. 1F). Over time, Yucatan minipig skin lightened and the shape and color of some of the CALMS changed as the animal grew in size with some markings becoming undetectable on photograph (Fig. 1C,D). Axillary/inguinal freckling was present in all *NF1*^*+/ex42del*^ animals at 4 months of age (Fig. S2).

### NF1 minipigs exhibit shortened stature and phenotypic trends in skeletal measurements

**Figure 2:**
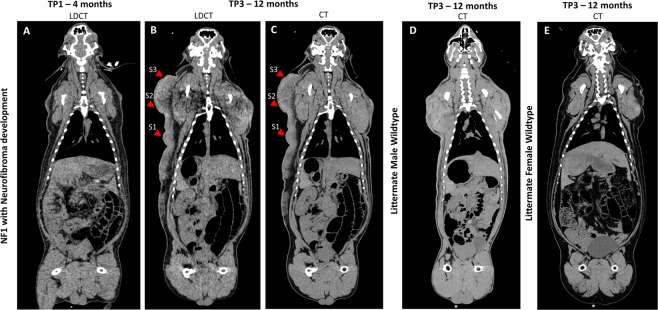
Subject with neurofibroma progression visualized with computed tomography (CT) (shown in coronal whole-body view) at (**A**) 4-months and (**B**) 12-months using the low-dose CT (LDCT) protocol and (**C**) 12-months using the standard-dose CT protocol. Note the granularity differences between CT and LDCT at time point 3 (TP3: 12 months of age), particularly in the areas (S1-S3) that were biopsied for histopathology. Comparison to litter-mate male wildtype with same scanning history (**D**) Demonstrates the ‘typical’ CT presentation of the lean body mass and hardening of uncastrated male pigs’ skin. Comparison to littermate female wildtype with same scanning history (**E**) shows the increased fat content in the intact female compared to intact boar. Window/level set to abdominal view.

At the three group imaging time points, the *NF1*^*+/ex42del*^ animals did not show significant differences in body weight compared to wildtype littermates (Mann-Whitney individual TP, p = 0.14–0.78; RM-ANOVA, p = 0.39). While NF1 minipigs trended (not significantly) to have slightly lower body weights, the mean difference in body weights between groups merged closer over time indicating minimal body mass difference between genotypes. The difference between the *NF1*^*+/ex42del*^ mean body weight and that of the wildtype was 4 kg at TP1, 2.5 kg at TP2 and 2.1 kg at TP3 (Table S1). The uncastrated boars tended to maintain a leaner body mass than their female littermates irrespective of genotype, which can be noted on imaging (Fig. 2D,E).

**Figure 3:**
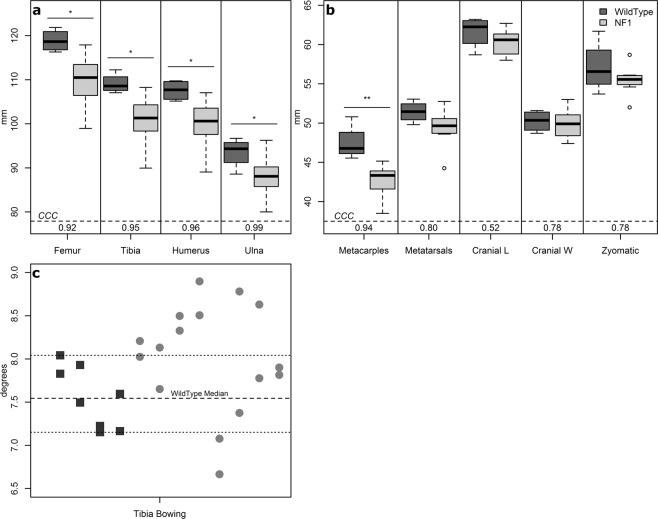
Bone measurements taken at 4-months of age indicate skeletal differences between *NF1*^*+/ex42del*^ (N = 8) and wildtype minipigs (N = 4). (**a**) long bone measurement boxplots with repeated measures’ CCC values, (**b**) additional long bone and cranial cavity measurement boxplots with repeated measures’ CCC values, (**c**) tibial bowing assessment plotted with dashed lines indicating range and median values for wildtype siblings. Definition of abbreviations: CCC – concordance correlation coefficient; L – length; W – width; NF1 – neurofibromatosis 1; mm – millimeters; *– p < 0.05; **– p < 0.01.

Human NF1 subjects often present with skeletal abnormalities including macrocephaly (24–45%), short stature (20–30%), and tibial bowing (3–4%)^3,37^. Skeletal measurements from CT imaging demonstrate shortened stature in *NF1*^*+/ex42del*^ minipigs at 4-months of age (Fig. 3). Bilateral symmetry was seen in the long bone measurements of both wildtype and *NF1*^*+/ex42del*^ subjects with the largest average difference of 1 mm, or a difference of ~1 voxel, between the left and right sides. The long bones of *NF1*^*+/ex42del*^ subjects were smaller on average (42.5–110 mm) with a higher standard deviation (2.1–6 mm) compared to their littermate wildtype controls (mean 47.4–119 mm; standard deviation 2–4 mm) (Fig. 3a). Measurement consistency assessed with Lin’s correlation coefficient (CCC) was highly repeatable for the long bones (femur, tibia, humerus, ulna, metacarpals) while metatarsals were less repeatable (CCC 0.78–0.80).

There was no statistical evidence of cranial differences in the *NF1*^*+/ex42del*^ subjects based on unilateral measurements, indeed the median and quartile cranial measurements for *NF1*^*+/ex42del*^ minipigs was smaller than their wildtype siblings (Fig. 3b). These three cranial measurements did suffer in repeatability (CCC = 0.52–0.78), indicating the need for more repeatable measures. Assessment of tibial bowing demonstrated six *NF1*^*+/ex42del*^ minipigs with a measurement of bowing higher than the range of the controls; bilaterally (N = 2) and unilaterally (N = 4) indicating a potential phenotype (Fig. 3c). One NF1 minipig had left leg bow angle of 8.9°, which is equal to one range deviation above the largest wildtype.

### NF1 minipigs develop neurofibroma which progress with longitudinal tracking

**Figure 4:**
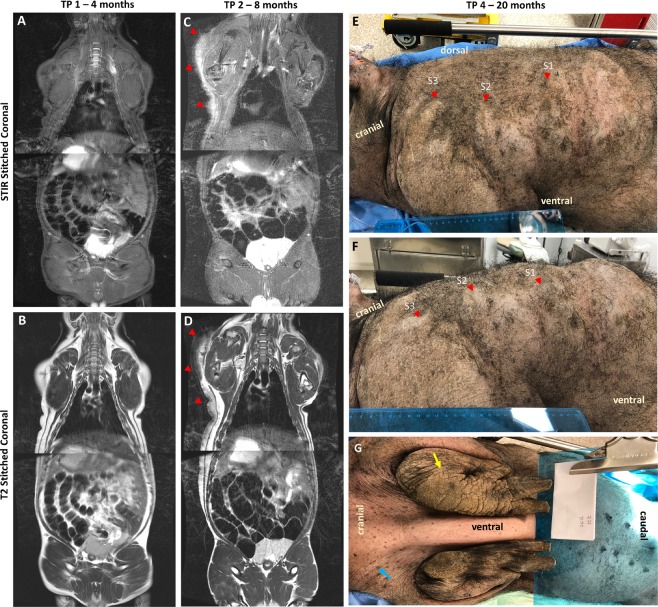
Imaging of neurofibroma subject. (**A-D**) Stitched stations of whole-body magnetic resonance (MR) Short Tau Inversion Recovery (STIR) and T2-weighted acquisitions for timepoint 1 (TP1: 4 months of age) (A-B: neurofibroma not detected) and timepoint 2 (TP2: 8 months of age) (C-D: neurofibroma detected). MR images are shown in mirror view with minipig’s left side on the left. Note the higher intensity region in TP2 STIR image with no comparable high intensity visualized at TP1. (**E-F**) Photographs taken of left-sided neurofibroma region at timepoint 4 (TP4: 20 months of age) with indicated regions of sampling (red). (**G**) Additional external findings in this subject included auxiliary freckling (blue) and edema on distal portion of the left foreleg (yellow) on the same side as the masses photographed in E-F.

**Figure 5:**
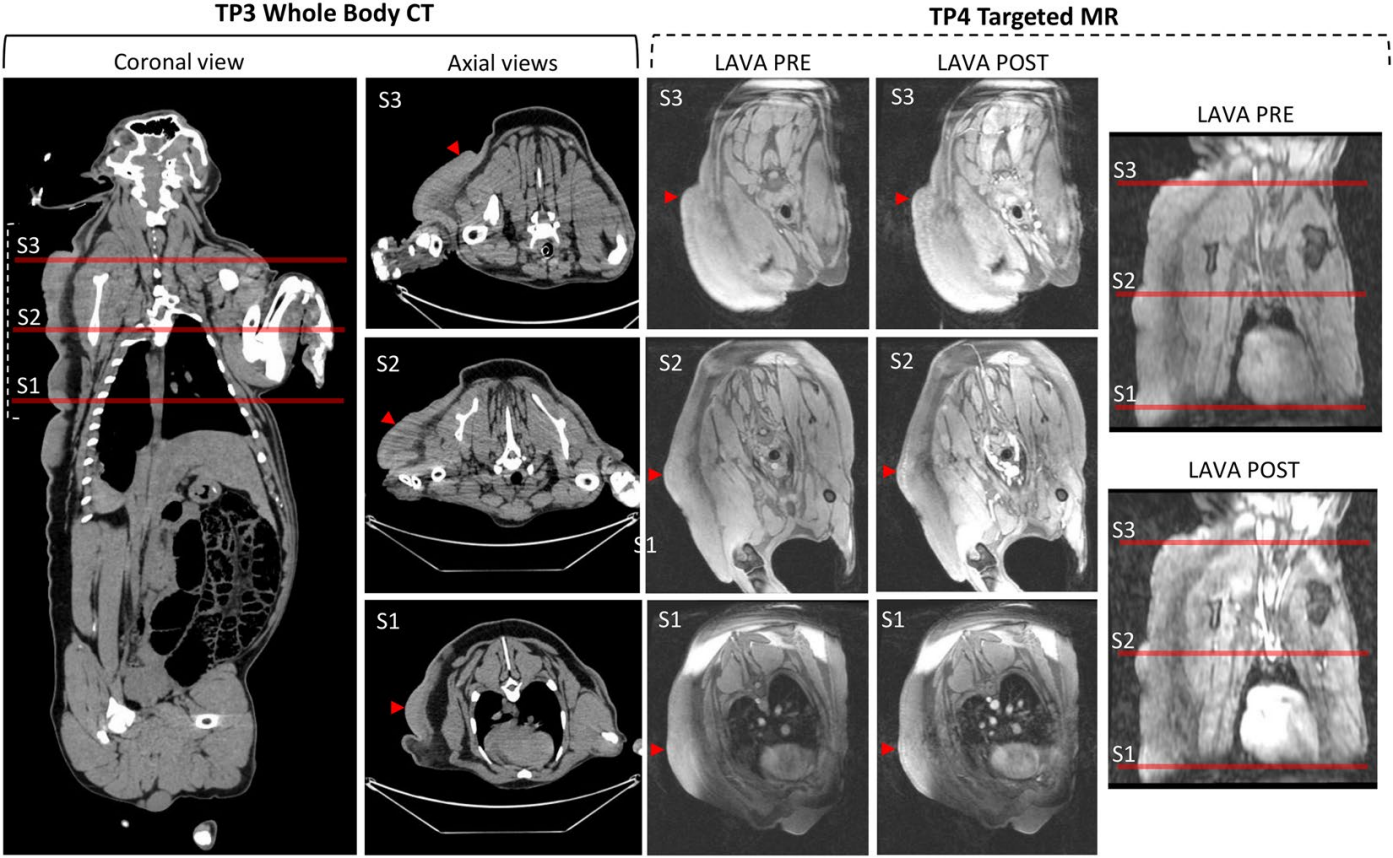
Final imaging timepoints (CT = TP3; MR = TP4) of neurofibroma. Timepoint 3 (TP3: 12 months of age) whole-body computed tomography (CT) scan shown in coronal view with three axial slices corresponding to three histological captures at necropsy (red, S1-S3), window/level is abdominal field. Timepoint 4 (TP4: 20 months of age) Targeted magnetic resonance (MR) acquisitions pre- and post- contrast agent for (S3 (two slices shown) and S2), window/level was kept consistent between pre-and post- contrast images.

Uncastrated boars often develop a tough outer layer of skin; in Fig. 2 we demonstrate a comparison to the littermate wildtype male and littermate wildtype female at TP3 (post-puberty CT) showing the imaging difference between the neurofibroma and the skin toughening. One out of eight *NF1*^*+/ex42del*^ minipigs developed a detectable neurofibroma. The unilateral lesion was first detected via imaging at the 8-month imaging time point (TP2) although, retrospective review of the 4-month time point (TP1) indicated potential early signs of abnormality (Figs. 2A–C and 4). In Fig. 4, we have indicated with red arrows the histopathology sampling areas on fused whole-body coronal screening stations (T2, STIR). Lateral asymmetry is demonstrated at 8-months with the appearance of the neurofibroma, while the 4-month scan indicates relative bilateral symmetry in the region. The neurofibroma was evident on CT at the 12-month time point (TP3) (Fig. 5) and appears to extend from the top of the left shoulder to the mid-abdominal flank region. By TP3 the tumor had developed both in diameter and protrusion; a final imaging time point (TP4) occurred prior to necropsy at 20 months (Fig. 5). Faint uptake of contrast can be seen in the TP4 scans with a granular appearance in MR 3D T1-weighted gradient recalled echo sequence (LAVA) sequence acquisition (Fig. 5).

**Figure 6:**
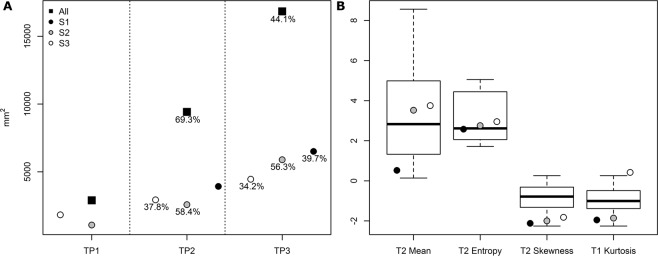
Image feature comparison between swine neurofibroma and plexiform neurofibroma’s from human subjects. (**A**) World Health Organization (WHO) tumor measurement criteria for suspected neurofibroma on 3D T1-weighted gradient recalled echo sequence (LAVA), post contrast magnetic resonance (MR); indicated percentage is growth from previous timepoint. See Table S2 for criteria raw measurements of longest and perpendicular axis. (**B**) Quantitative imaging characteristics extracted from the regions of interest (ROIs) (S1, S2, S3) compared to those seen in the retrospective human cohort.

Measurements of the neurofibroma regions were made using the World Health Organization (WHO) criteria on the LAVA post contrast scans (Fig. 6A); WHO is taken as the product of the two longest perpendicular measurements of a tumor with progressive disease defined as ≥25% increase in one lesion^38^. All three regions grew between imaging timepoints in both longest axial diameter and perpendicular cross length (Table S2) and at a rate >25% indicating progressive disease. The S3 region grew more in the perpendicular region (deeper) than the other two regions which had more expansive growth in longest diameter. The perpendicular growth can be attributed in part by the overall growth of the minipig in general as the longest axial diameter tended to align tangential to the thorax.

**Figure 7:**
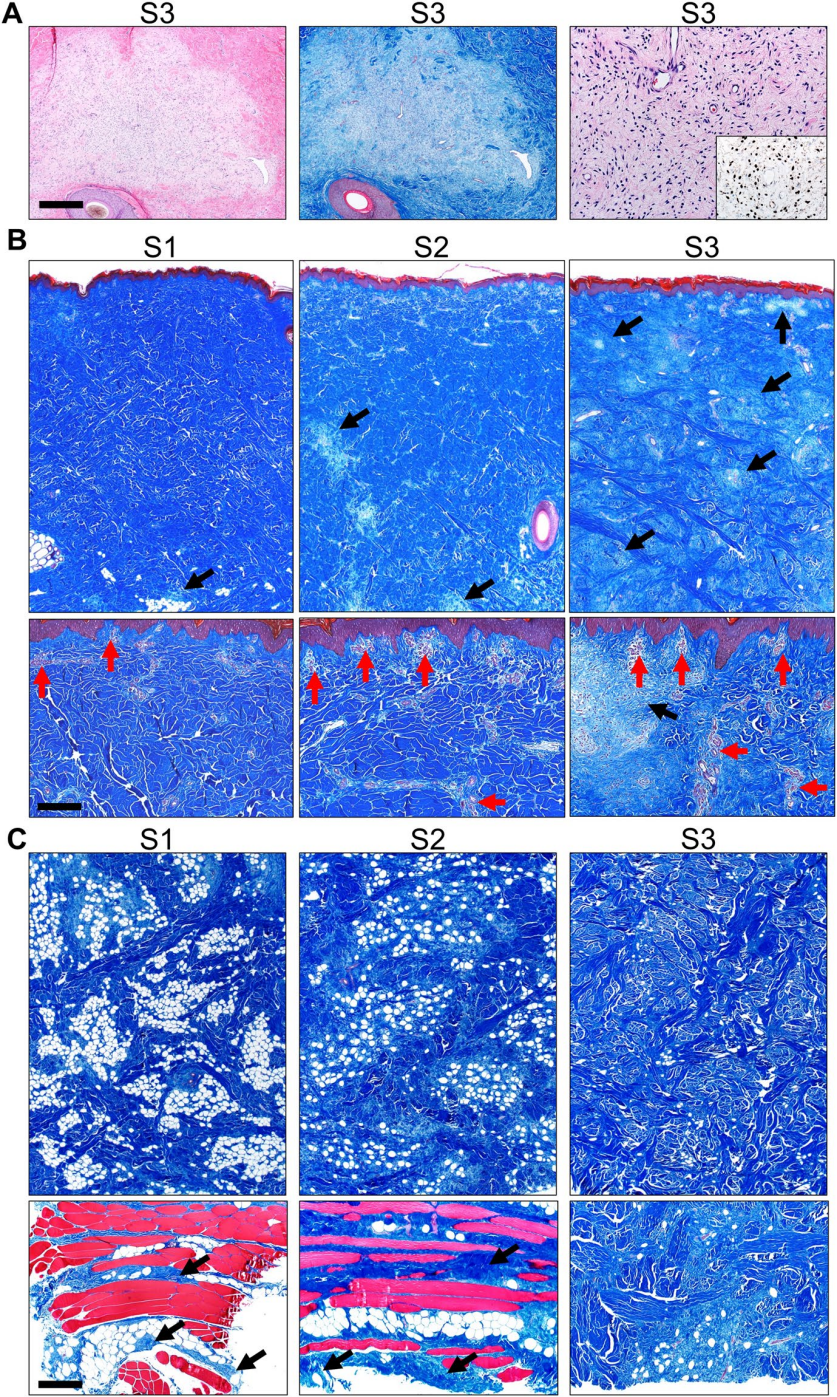
Histopathology of tumor tissues collected at different stages of growth labeled as S1 (least mature), S2, and S3 (most mature). (**A**) Sections of S3 dermis with multifocal effacement by tumor tissue in HE (left) and MT stains sections (middle), bar = 485 µm. The neurofibroma was composed of low-moderate cellularity (HE, right) and scattered S100 positive cells (inset, right), bar = 98 µm. (**B**) Skin tumor sections containing epidermis and dermis in S1-3 samples, MT stained sections, bar = 966 and 194 µm (top and bottom panels, respectively). Neurofibroma tissues (black arrows) progressively expanded into and effaced much of the dermis (see S1-3). Neurofibroma tissues extended to the upper limits of the dermis but did not cross the basement membrane into the epidermis. Neurovascular networks (red arrows) were increased in prominence from S1 to S3, especially in the upper dermis. (**C**) Skin tumor sections from the subcutis in S1 toS3 samples, MT stain, bar = 966 and 483 µm (top and bottom panels, respectively). *Top panels*: From S1 to S3, neurofibromas had a more prominent fibrous appearance (i.e. increased collagen deposition evidence by increased blue staining) that also effaced normal adipocytes (white circular structures). *Bottom panels*: In the subcutis, cutaneous trunci muscles (see red muscle in bottom panels) had evidence of infiltration by the neurofibroma (arrows) in samples S1-2. In S3 (bottom right), the collagen-rich (blue staining) neurofibroma extended to the deep excision margins, but cutaneous trunci muscle was not detected.

At necropsy, tumor tissue was excised from three regions based on clinical appearance of the tumor, and labeled S1 (least mature), S2 and S3 (most mature). The tumors sections were relatively well-defined along the deep border and excised easily. At histopathology, each of the skin tumor sample sites had evidence of neurofibroma consistent with what was previously described in the model as diffuse cutaneous neurofibroma (Fig. 7)^22^. Tumor samples had progressive changes that correlated with the duration of the tumor including: increased prominence of the neurovascular networks noted by increased vascular structures, progressive infiltration and effacement of the dermis/subcutis by tumor, and maturation of neurofibroma composition from myxoid to more fibrous appearance.

### NF1 Minipigs develop neurofibroma with quantitative imaging features similar to human subjects

Increasing change in signal intensity post-contrast was seen between tumor regions S1 (10.8%) and S2 (12.8%); the S3 region of interest (ROI) had the largest uptake of contrast with a percentage signal intestisty change (_%_SIΔ) of 13.6%. The two adjacent slices to each ROI were assessed with differences in _%_SIΔ between <0.1% and 0.3%. In general, an increase in signal intensity indicates permeable blood vessels characteristic of tumor tissue – specifically where the gadolinium contrast has leaked into the extracellular matrix^39^. Prior work has found higher rates of contrast enhancement in neurofibromas when compared to malignant peripheral nerve sheath tumors in human subjects with NF1, and further that this was negatively correlated with the degree of microscopic necrotic area^11^. Quantitative imaging characteristics were extracted from the three ROIs (S1, S2 and S3) and compared to previously published values for plexiform neurofibroma assessment (Fig. 6B) which showed the values for each of the three ROIs followed trends seen in a retrospective human cohort^40^.

### Systemic imaging of minipigs allows for investigation of other findings

In our cohort of 8 *NF1*^*+/ex42del*^ minipigs we did not see indication of optic glioma or optic chiasm tumor (Fig. S3A). Assessment of the brain did not demonstrate presence of “unidentified bright objects” which was present in the F0 boars^22^ and have been associated in NF1 humans with cognitive dysfunction (Fig. S3B)^22,41–46^. Two of the *NF1*^*+/ex42del*^ subjects exhibited deep shoulder lesions that could not be identified upon necropsy (Fig. S3C). These lesions were consistent with a previous injury or preanesthetic delivery prior to image acquisition, as has been sometimes seen. A finding previously undescribed in MR and CT imaging was the presence of an enlarged accessory sex gland in uncastrated males (Fig. S3D) which appears bright on STIR, isointense on T2, and similar to soft-tissue on CT^47^. Optic gliomas are a manifestation of NF1 occurring in an estimated 15% of children with the disease. Measurement of the optic nerve and sheath indicated no significant difference (p > 0.19) between *NF1*^*+/ex42del*^ and wildtype (Fig. S4**)**.

## Discussion

We longitudinally studied an NF1 porcine model (*NF1*^*+/ex42del*^) for the first 12-months of life to evaluate phenotype development, track disease progression, and provide a comparison to human subjects. Through systematic tracking we have shown that an *NF1*^*+/ex42del*^ minipig model develops phenotypic characteristics [1] CALMs, [2] axillary/inguinal freckling, [3] shortened stature, [4] tibial bone curvature, and [5] histological variations in neurofibroma growth. With littermate wildtype controls we confirmed that these findings were exclusive to minipigs with NF1 genotype. The breadth of common NF1 phenotypes presented in this cohort indicates minipigs as a translational correlate to the diversity seen in the human presentation of NF1. Through this study we outlined a set of imaging protocols that allow for the longitudinal tracking of phenotypes and demonstrated the effectiveness of tracking on a minipig developing neurofibroma. The ability to longitudinal track neurofibroma progression with non-invasive medical imaging modalities common to human subjects is powerful for preclinical applications such as drug response assessment.

The series of imaging protocols utilized for this study were guided by recommendations from the REiNS International Collaboration which indicates that MR imaging acquisition should be optimized for tumor type/location and recommends both whole-body imaging and the STIR protocol for visualization of peripheral nerve sheath tumors^48,49^. However, it was noted in Dombi *et al*. that some clinically relevant tumors may not have good tissue definition on MR imaging with the accompanying Fig. 3a,b of that manuscript displaying a superficial flank plexiform neurofibroma^48^. The development of neurofibroma in a minipig model allows for robust comparison of protocols and modalities which is not possible in human subjects. We recorded the development and growth of a neurofibroma in a minipig model over the course over a 20-month period with four imaging time-points consisting of screening MR, targeted MR, and whole-body CT. We demonstrated detection of neurofibroma with LDCT and with additional testing this could suggest LDCT as a viable option for whole-body screening with reduced radiation exposure over standard CT, and as a fast acquisition (not requiring anesthesia) alternative to MR.

We performed an extensive CT-based skeletal measurement assessment in our matched cohort. Short stature is common among NF1 adults with 20–30% below the 3^rd^ percentile in height^37^. We found statistically smaller long bones (femur, tibia, humerus, ulna, metacarpals) in the *NF1*^*+/ex42del*^ minipigs compared to their wildtype siblings. Previous NF1 minipig studies found no difference in stature using external measurement of length and height^23^. However, prior studies have recorded strong association between femur measurement and human stature in adults^50^ and children^51^ and have been used as a surrogate measure of growth differences between rats^52^ and pigs^53^. Hence, we conclude that the significant difference in multiple bones of the *NF1*^*+/ex42del*^ minipigs is indicative of the short stature that is associated with NF in human patients. Interestingly, the metatarsals were the only long bones to not be statistically smaller in the *NF1*^*+/ex42del*^ minipigs; recently, Faria *et al*. described a potential new phenotype of NF1 in which the second toe of subjects is elevated and super-positioned on the third toe, visual assessment of minipig metatarsal arrangement did not demonstrate evidence of this characteristic^54^. Tibial bowing is a rare but distinctive phenotype in NF1 (3–4%)^37^, we report quantitative results of increased bilateral and unilateral bowing in NF1 minipigs out of the range of wildtype sibling controls, indicating a potential phenotype. Isakson *et al*. described bilateral tibial dysplasia narrowing in two cloned boars using qualitative radiograph assessment but did not note other skeletal abnormalities in the F1 cohort^23^. Weight was not statistically significantly different between *NF1*^*+/ex42del*^ and wildtype minipigs at any of the three study time points (4, 8 and 12 months of age). Previous studies have indicated a reduced weight NF1 minipigs using a small selected cohort (N = 3)^23^.

The skin of the Yucatan minipig has previously been shown to have similar characteristics to human skin including epidermal thickness and melanin assessment^55^. This was confirmed with the presence of NF1 dermal manifestations including CALMs and axillary/inguinal freckling in all *NF1*^*+/ex42del*^ subjects in this cohort. Over 99% of humans with NF1 present with CALMs ranging from 0.5 cm to greater than 20 cm in diameter^56^. Here, through longitudinal tracking of CALMs we found the minipig presentation showed similarity in size (1.0 cm–5.0 cm). One *NF1*^*+/ex42del*^ subject in our cohort presented with a giant CALM (>20 cm in diameter) along the left side; giant CALM is a rare NF1 manifestation reported in cases of segmental sporadic NF1 mutation with associated with plexiform neurofibroma and MPNST^57–59^. As the minipig subjects aged, the skin tended to lighten making it difficult to visually inspect the CALMs; this is in contrast to the human population where increased skin pigmentation due to sun exposure is common^56^.

There are currently two NF1 minipig published models: the Yucatan *NF1*^*+/ex42del*^^22,34^ and the Ossabaw *NF1*^p.Arg1947* 23^. The two models have some similarities and variations which will benefit the research community and provide the mechanism for more individualized choices in phenotypical presentation. In our previously published Yucatan *NF1*^*+/ex42del*^ cohort we demonstrated imaging of neurofibromas in three male NF1 pigs that also presented with CALMs^22^. In this study, we have tracked individual CALM from 4 to 12 months of age - correlating to prepubescence through early adulthood in humans – finding that CALMs in minipigs across this time period are stable is size. Further, we have demonstrated presence of axillary/inguinal freckling through the 12 months of age. Unlike our previous cohort of *NF1*^*+/ex42del*^ swine, the animals presented here are the offspring of cloned boars heterozygous for the *NF1* exon 42 deletion bred to wildtype sows^22^. There is some evidence to suggest that cloned (F0) animals may exhibit weakened immune systems and other abnormal phenotypes in adulthood, resulting in divergent health outcomes and irregular behavior^60,61^. In this study we have excluded those potential effects by using naturally bred first generation (F1) minipigs. Isakson *et al*. recently reported in an Ossabaw minipig model the development of CALMs (F0, F1), freckling (F1), Lisch nodules (F0), dermal neurofibroma (F0, F1), a glioma-like lesion in optic chiasm (F1), and tibial narrowing (F0)^23^. In this study’s Yucatan F1 cohort we found no evidence of optic chiasm lesion.

This study has limitations. While this is the first systematic longitudinal study of a cohort of *NF1*^*+/ex42del*^ animals with littermate wildtype control animals, the study contained a limited number of animals (N = 12). As some clinically important NF1 phenotypes are rare in the human population, this small animal cohort may not fully represent the spectrum of phenotypes present in the *NF1*^*+/ex42del*^ swine model. We did not systematically screen this cohort of animals for Lisch nodules or cognitive impairment. Abnormal curvatures of the spine are potential skeletal abnormality associated with NF1, however, in our study we were not able to differentiate imaging assessed spinal curvature from positional distortion in our anesthetized, prone minipigs. It is possible this could be assessed in future studies by incorporating a different positioning approach for image acquisition. This study was designed to systematically image the animal cohort for the first 12 months of age for early characterization and progression over this time-period (early childhood and adolescence). The one *NF1*^*+/ex42del*^ animal that presented with a neurofibroma within 12 months was necropsied at 20 months. Hence, we cannot determine if further members of the cohort would have developed neurofibromas after 12 months of age - note in our prior study of the F0 cohort neurofibromas were discovered between the ages of 11 and 17 months of age^22^. Due to the yet unknown role of hormones on NF1 phenotype development, the male animals in this study were uncastrated which can present some challenges for longitudinal housing and scientific study. Uncastrated boars have increased aggression and sexual behaviors, along with physical characteristics such as tusk growth and thickened subcutaneous layer of tissue (aka shoulder plate). These characteristics can require individual housing and increased caution (and personnel) for transport, anesthesia administration, and recovery.

In conclusion, *in-vivo*, clinical equivalent medical imaging protocols including CT and MR were used to systematically evaluate early development of NF1 related phenotypes in a matched cohort of male and female *NF1*^*+/ex42del*^ animals with littermate wildtype control animals. In this study we found penetrant presentation of shortened stature and tibial curvature. Typical skin manifestations of NF1 including CALMs and axillary/inguinal freckling were identified as stable throughout the *NF1*^*+/ex42del*^ minipig life-course. One out of eight *NF1*^*+/ex42del*^ animals developed a neurofibroma which progressed over time, paralleled to imaging characteristics of human neurofibromas, and demonstrated differential maturation on histopathology. This *NF1*^*+/ex42del*^ Yucatan minipig model presents a powerful tool for optimization of medical imaging protocols and preclinical therapeutic evaluation.

## Materials and Methods

### Animal procedures

All procedures were approved by the Institutional Animal Care and Use Committees (IACUC) of the University of Iowa and Exemplar Genetics (Coralville, Iowa) and all procedures were performed in accordance with relevant guidelines and regulations. 12 Yucatan minipigs (6 female, 6 male), 8 heterozygous for NF1 (*NF1*^*+/ex42del*^) and 4 littermate wildtypes, were systematically imaged at 4-, 8-, and 12-months of age using both CT and MR imaging. Animals were anesthetized using either a combination of midazolam (0.6 mg/kg) and ketamine (2.2 mg/kg) or telazol (2.2–4.4 mg/kg), ketamine (1.1–2.2 mg/kg), and xylazine (1.1–2.2 mg/kg). Anesthesia was maintained throughout animal preparation and imaging using isoflurane (1–5%). Each pig was intubated with a balloon-cuffed endotracheal tube (6–8 mm) and mechanically ventilated using a Primer SP MRI-Compatible Veterinary Anesthesia Ventilator (DRE Veterinary). Animals were ventilated under 100% oxygen with a respiratory rate of 16-20 breaths per minute, 5cmH_2_O positive end expiratory pressure, and a tidal volume of approximately 10 mL/kg. Physiological monitoring was performed using electrocardiogram, oxygen saturation (SP-O2), and end tidal carbon dioxide pressure (ET-CO2). Ventilation parameters were modified when necessary to maintain a heart rate between 80–100 beats per minute and an SP-O2 of 95–100%. Prior to imaging, animals were placed in the prone position with their hind limbs extended behind them and their forelimbs fully adducted to their sides to provide stability. Mechanical ventilation was maintained throughout imaging; no breath holds were performed.

### Imaging procedures

A longitudinal imaging study was devised with three proposed imaging timepoints at 4-months (TP1), 8-months (TP2), and 12-months (TP3) of age (Fig. S1). Scans were systematically reviewed at each timepoint by a radiologist (T.S.S.) specializing in pediatrics and neuroradiology using a structured method of reporting. Review was performed unblinded to known genotype (*NF1*^*+/ex42del*^ /wildtype) and preceding timepoint scans were provided for longitudinal assessment concordant with clinical practice.

A non-contrast enhanced, full body screening CT was acquired at TP1 and TP3 using a Siemens SOMATOM Force CT scanner (Siemens Healthcare, Forcheim, Germany) or a GE Discovery MI PET/CT (GE Healthcare, Waukesha, WI). The following standard dose parameters were used for acquisition: Spiral, 70 kV, 208 ref. mAs (tube current modulation on), pitch 1.9. Data was reconstructed as 0.75 and 2 mm slice thicknesses using iterative reconstructions (Qr40, ADMIRE level 5 or STANDARD, ASiR 100%). A subsequent equivalent scan was acquired at a lower radiation dose level with the same parameters but with a 20-reference mAs.

MR imaging was performed at all timepoints using a 3.0 T General Electric Discovery MR750w. Four separate protocols were used for imaging: head, whole body, spine, and targeted. The developed protocols drew from whole-body screening recommendations and typical clinical practice^48,49^. The targeted protocol was used only when an unexpected or abnormal finding was identified during one of the prior three protocols. Table 1 lists the scans of each protocol and their parameters. Head scans were completed using a 32-channel head coil. Targeted scans were completed using the 8US TORSOPA abdominal coil.

**Table 1:** Magnetic Resonance (MR) imaging parameters.

Protocol	Scan	Coil	Voxel Resolution	FOV	TR	TE	Flip Angle
Head	Sag FSPGR BRAVO	Head	1.0 × 1.0 × 1	256 × 256	8.46	3.22	12
	Sag T2 Cube	Head	1.0 × 1.0 × 1	256 × 256	3000	90.52	90
	FLAIR	Head	0.47 × 0.47 × 1	512 × 512	5000	90.10	90
Whole Body	Station 1 STIR	Body	1.0 × 1.0 × 1	512 × 512	9500	45.08	111
	Station 1 T2	Body	1.0 × 1.0 × 1	512 × 512	3800	104.61	111
	Station 2 STIR	Body	0.82 × 0.82 × 5	512 × 512	9500	45.02	111
	Station 2 T2	Body	0.82 × 0.82 × 5	512 × 512	4200	104.27	111
Spine	Station 1 Spine	Body	0.78 × 0.78 × 4	512 × 512	9500	44.46	111
	Station 2 Spine	Body	0.86 × 0.86 × 4	512 × 512	9500	44.35	111
Targeted	Ax T2 FS PRE	Abdominal	0.78 × 0.78 × 5	512 × 512	4000	104.28	111
	Ax LAVA-PRE	Abdominal	0.78 × 0.78 × 5	512 × 512	4.04	1.89	12
	Ax LAVA -POST	Abdominal	0.78 × 0.78 × 4	512 × 512	4.08	1.89	12

*Sag =* *Sagittal plane, FSPGR =* *Fast Spoiled Gradient Echo, BRAVO =* *Brain Volume Imaging, FLAIR =* *Fluid Attenuated Inversion Recovery, STIR =* *Short Tau Inversion Recovery, LAVA =* *Liver Acquisition with Volume Acceleration, Ax =* *Axial plane, PRE =* *pre-contrast administration, POST =* *post-contrast administration, FOV =* *Field of View, TR =* *Repetition Time, TE =* *Time to Echo*.

### Clinical measurements and recording

At each imaging timepoint, minipig weight and exterior clinical indications were recorded. With the anesthetized minipig positioned laterally, photographs were taken the right and left sides. Additional photographs of encountered phenotypes were taken including CALMs, axillary and inguinal freckling, and exterior indications of neurofibroma. For each minipig exhibiting CALMs, the diameter of the largest marking on each side was measured and tracked for change across the imaging timepoints. A description of this method is included as Supplemental Text.

### Skeletal measurements

Using the CT scans from TP1, skeletal measurements were taken with tools available in 3D Slicer (https://www.slicer.org/) to identify any developmental abnormalities typically seen in NF1 patients. A full description with images of these CT based skeletal measurements is included as Supplemental Text. To summarize, length measurements were made of the long bones on both lateral sides including the femur, tibia, humerus, ulna, metacarpals, and metatarsals. Presence of anterolateral tibia bowing was assessed using an angle measurement previously described in an NF1 mouse model^62^.

### Optic nerve measurements

Longitudinal measurements of the optic nerves from head MR images were obtained using a method previously described^63^. A full description with images of measurements is included as Supplemental Text. To summarize, measurements of the diameter of the optic nerve with and without the nerve sheath were taken from the axial plane of the T2 CUBE datasets. These diameters were systematically measured at 3 mm behind the ocular globe in the image slice that provided the clearest view of the nerve.

### Histopathology neurofibroma assessment

For minipig tumor development assessment, at necropsy tissue was taken from identified sites of interest (Fig. 2). At each site, tumor tissue was excised and samples collected from epidermal/dermal region as well as the subcutis and deep tumor margins. Tumor tissues were fixed (10% neutral buffered formalin x 5–7 days), processed, paraffin-embedded, sectioned (~4 µm) and stained for hematoxylin and eosin (HE) or Masson’s Trichrome (MT) stains. S100 immunohistochemistry was performed as detailed previously^64^. Tissues were examined by a veterinary pathologist following principles of reproducible histopathological examination and scoring^65^. As the tissues came from the same animal and were used principally for comparative descriptions, masking of the samples was not necessary.

### Imaging-based neurofibroma assessment

The corresponding regions for histopathology were assessed in the T2 FS, LAVA Pre- and LAVA Post- contrast scans for quantitative imaging characteristics. Measurements of the neurofibroma regions were made using the World Health Organization (WHO) criteria on the LAVA post contrast MR scans; WHO is taken as the product of the two longest perpendicular measurements of a tumor, progressive disease is defined as ≥25% increase in one lesion^38^. Regions of interest (ROI) corresponding to biopsy zones were extracted from axial slices as regions with consistent area of 1400 square pixels. Percentage signal intensity change (_%_SIΔ) was extracted from the ROIs as previously described^66^. Additional quantitative imaging features (T2 Mean, T2 Entropy, T2 Skewness, and LAVA-Pre Kurtosis) were extracted and compared to published values on retrospective human clinical subjects with benign plexiform neurofibromas^40^.

### Statistical analysis

A two-sample Wilcoxon rank sum (Mann-Whitney) test was performed between *NF1*^*+/ex42del*^ and wildtype minipig weight and skeletal measurements^67^. For the ten subjects with three timepoints, subject weight growth across time was compared using repeated measures analysis of variance (RM-ANOVA) following assessment of Mauchly’s sphericity test with two degrees of freedom^68^. An alpha value of 0.05 was used to establish significance on a statistical level. Repeated skeletal measurements were compared using Lin’s concordance correlation coefficient (CCC) with a value ≥0.90 considered repeatable^69^.

## Supplementary information

Supplementary information.

## Data Availability

The datasets generated during and/or analyzed during the current study are partially available in the Synapse (Sage Bionetworks) Children’s Tumor Foundation repository; https://www.synapse.org/#!Synapse:syn6135075/wiki/470486. The datasets generated during and/or analyzed during the current study that are not available via Synapse, are available from the corresponding author on reasonable request.
