# Supplementary material for: Longitudinal phenotype development in a minipig model of neurofibromatosis type 1
Source: Sci Rep. 2020 Mar 19;10:5046. doi: 10.1038/s41598-020-61251-4 (PMC7081358; doi:10.1038/s41598-020-61251-4)
Supplement: Supplementary file 1 — Supplementary information. [file 41598_2020_61251_MOESM1_ESM.docx]

**SUPPLEMENTAL MATERIALS**

To accompany the publication Uthoff et al. “Systematic longitudinal evaluation of phenotype development in a swine model of neurofibromatosis 1”

**Figure S1** **– Flowchart of study course.** Indicated with icons are the timing of systematic recording of phenotypes not necessarily the onset of phenotypes – for example, café au lait markings were present on NF1 subjects from birth but were systematically recorded and tracked from 4-months of age. The study cohort was comprised of littermates from 4 litters sired by three different NF1 boars and selected to provide a balance between gender across NF1 and wildtype (WT) animals.

**Supplemental Text**

**Measurement of Café au Lait Markings**

Digital photographs were obtained with scaling of both right and left sides of each minipig at each imaging timepoint (4-, 6-, and 12-months of age). Selected café au lait markings (CALMs) for the NF1 minipigs were measured across timepoints (**Figure A**). As some CALMs became undetectable through photograph, only markings that were well visualized throughout the three timepoints were considered for measurements. For each timepoint, the largest macule on each side was measured and recorded.

**Figure A** - Example of the café au lait macule measurements.

**Measurements of Skeletal Abnormalities**

CT images of 8 pigs heterozygous for the NF1 gene and 4 wildtype littermate controls were obtained with slice thicknesses of both 0.75 and 2.0 mm with a 70 kV peak and 208mAs. All animals were placed in prone position with their posterior limbs spread and their anterior limbs adducted to their sides. Measurements from these images were taken with 3D slicer software tools (<https://www.slicer.org/>). The skeleton was extracted through thresholding of the CT image at bone-level intensity of +300 HU (**Figure B**).

As height is largely determined by the length of the long bones, measurements of the femur, tibia, humerus, and radius were of particular interest. Femoral length was measured from the greater trochanter to the lateral epicondyle, both of which are visible from lateral views of the animal.

Tibial length was measured from the medial condyle to the medial side of the inferior articular surface, which was easily visible from an anterior view of the skeleton. Tibial bowing was measured as described in a Wang et al. (Figure 3-F in *Wang 2011 Hum. Mol. Genet*.) who used the measurement of anterolateral bowing in a mouse NF1 model^1^. To measure this abnormality, the greatest distance from the midline of the bone at the base of the tibial tuberosity (MB) to the lateral most portion of the bone was recorded (LB). The angle (in radians) was calculated by taking the inverse cosine of these measures = cos^-1^(MB/LB).

The ulna was observed with a dorsolateral view of the skeleton; in this position the length can be measured from the olecranon to the point where the radius articulates with the carpals (**Figure C, left**).An anteromedial view of the skeleton provided enough detail to measure the length of the humerus from the peak of the major (lateral) tuberosity to the end of the lateral epicondyle (**Figure C, right**).

The metatarsals and metacarpals of the minipigs were measured from their proximal area of articulation with the carpals or tarsals to their distal most point of contact with the phalanges.

In order to measure cranial differences in the porcine model, the distance from the base of the zygomatic arch to the frontal bone was recorded (**Figure D**). This measurement was easily accessible via a lateral view of the model. As an additional measure, the greatest diameter of the frontal bone from a dorsal view of the animal was recorded. By altering the opacity of the model (between 0.2 and 0.3) the cranial cavity was visible. This alteration allowed for the measurement of the greatest dimension of the cranium along both the sagittal and axial planes.

Figure B: Example measurements of skeletal features including zygomatic, femur, metacarpals, humerus, ulna, tibia, and metatarsals.

**Measurements of Optic Nerve**

MR images of the head were obtained for all minipigs at each imaging timepoint. Diameter measurements for the optic nerve of both the right and left eyes, including and excluding the optic nerve sheath, were taken with the OsiriX software on the axial plane/fat-suppressed T2-weighted images. As previously described in Geeraerts et al. to have good repeatability, the diameters were measured at 3 mm behind the ocular globe in the image slice that provided the clearest view of the nerve (**Figure C**)^2^.

**Figure C –** Example minipig optic nerve measurements performed on the left ocular region of a male NF1 subject’s at time point 2 (TP2: 8 months of age). Orange arrow indicates measurement of the nerve with sheath (4.52mm), blue arrow indicates measurement of the nerve without sheath (1.98mm), the green line shows distance these measurements were taken from the optic globe (~3mm).

**Figure S2: Café au lait macules and freckling. [a]**- Photograph of NF1 minipig at time point 1 (TP1: 4 months of age) positioned supine showing inguinal freckling (yellow) alongside a café au lait macule (CALM) (red). [b] Multiple CALMs were present on each of the eight NF1 subjects systematically evaluated in the study. The CALMs could be tracked over time (red arrows) from 4 months of age (TP1) to 12 months of age (TP3). All four wildtype (WT) subjects demonstrated a clear absence of CALMs.

**Table S1: Weight comparison between NF1 and wildtype (WT) cohort over time.** While a trend to lower body weight is seen in NF1 compared to WT littermate subjects, this trend did not reach statistical significance on single-timepoint assessment (Mann-Whitney) or across time-points (RM-ANOVA, p = 0.38).

| Genotype | ID# | Sex | Weight (kg) | | | | |
| --- | --- | --- | --- | --- | --- | --- | --- |
|  |  |  | TP1 | TP2 | | TP3 | |
| WT | 4595 | F | 29 | | 36 | | 67 |
|  | 7174 | F | 22 | | 44 | | 60 |
|  | 4558 | M | 22 | | 37 | | 57 |
|  | 7175 | M | 24 | | 37 | | 45 |
|  | Mean (SD) | | 24.3 (3.3) | | 38.5(3.7) | | 57.3(9.2) |
| NF1 | 4592 | F | 20 | | 40 | | 56 |
|  | 7171 | F | 23 | | 39 | | 50 |
|  | 7173 | F | 26 | | 42 | | 54 |
|  | 4559 | M | 22 | | 39 | | 60 |
|  | 4560 | M | 19 | | 34 | | 57 |
|  | 7177[1] | M | 22 | | 34 | | 54 |
|  | 7179[2] | M | 17 | | - | | - |
|  | 4563[2] | F | 13 | | 24 | | - |
|  | Mean (SD) | | 20.3(4.0) | | 36.0(6.1) | | 55.2(3.4) |
| Mann-Whitney p-value | | | 0.14 | | 0.78 | | 0.45 |

*[1] – 7177 had an additional timepoint (TP4) weighing 85 kg*

*[2] – 7179 and 4563 were necropsied prior to study completion, subjects were not included in statistical testing*

**Table S2** – World Health Organization (WHO) criteria raw measurements - longest axis (LA) and perpendicular axis (PA) - from the three regions of interest across the three timepoints (TP2-TP4) when tumor was captured on imaging.

| Tumor Region | TP2 (mm) | | | TP3 (mm) | | | TP4 (mm) | | |
| --- | --- | --- | --- | --- | --- | --- | --- | --- | --- |
|  | *LA* | | *PA* | *LA* | *PA* | | *LA* | *PA* | |
| S1 | - | - | | 98 | | 40 | 130 | | 50 |
| S2 | 67 | 16 | | 78 | | 33 | 151 | | 39 |
| S3 | 65 | 28 | | 77 | | 38 | 89 | | 50 |

**Figure S3 -** Other study findings on imaging. a) Optical nerve imaging in female NF1 and female wildtype sibling showed no evidence of optical glioma. b) Optic chiasm and brain imaging on female NF1 and female wildtype sibling showed no evidence of optical chiasm tumor or other brain lesions. c) Unidentified shoulder lesion in male NF1 (yellow) shown in coronal whole-body Station 1. d) Large auxiliary sex gland evident (red) in intact males on magnetic resonance (MR) and computed tomography (CT) imaging. Coronal view of Station 2 Whole body screening with STIR and T2-weighted at time point 1 (TP1: 4 months of age) for both pigs. CT at time point 3 (TP3: 12 months of age) is shown for both animals in abdominal window/level.

**Figure S4: Measurement of the a) optic nerve and b) optic nerve sheath.** No statistical difference was seen between NF1 and controls (p >0.19).

**References**

1. Wang W, Nyman JS, Ono K, et al. Mice lacking Nf1 in osteochondroprogenitor cells display skeletal dysplasia similar to patients with neurofibromatosis type I. *Human molecular genetics* 2011;20(20):3910-24. doi: 10.1093/hmg/ddr310 [published Online First: 2011/07/16]

2. Geeraerts T, Newcombe VF, Coles JP, et al. Use of T2-weighted magnetic resonance imaging of the optic nerve sheath to detect raised intracranial pressure. *Critical care (London, England)* 2008;12(5):R114. doi: 10.1186/cc7006 [published Online First: 2008/09/13]
